# Editorial: Coronavirus disease (COVID-19): Psychoeducational variables involved in the health emergency

**DOI:** 10.3389/fpsyg.2022.961261

**Published:** 2022-09-26

**Authors:** Jesus de la Fuente, Douglas F. Kauffman, Michel S. Dempsy, Yashu Kauffman

**Affiliations:** ^1^Department of Theory and Methods Research in Education and Psychology, School of Education and Psychology, University of Navarra, Pamplona, Spain; ^2^Department of Psychology, School of Psychology, University of Almeria, Almería, Spain; ^3^School of Clinical Medicine, Medical University of the Americas-Nevis, Devens, MA, United States; ^4^Boston University, Boston, MA, United States; ^5^Commonwealth Corporation, Boston, MA, United States

**Keywords:** coronavirus disease (COVID-19), psychoeducational variables, health emergency, emotional psychological impact, psychological support

This monograph has allowed us to present a psychoeducational view of the effects of the COVID-19 pandemic. We confirm here that research in education contributes its own evidence and specific models for identifying this problem.

The first paper gives us a general overview and review of the problem (Cachón-Zagalaz et al.). Next, a joint editorial paper presents a novel theoretical model that provides for a purely psychoeducational analysis of the pandemic (de la Fuente, Kauffman, et al.).

A second group of articles presents the psychological and emotional impact of the COVID-19 pandemic in students of different ages. Several papers address university students (Fernández-Castillo). Next, other research studies address the preuniversity level, in childhood and adolescence (Andrés-Romero et al.; Berasategi et al.; Martarelli et al.; Valadez et al.; Zaccoletti et al.). Finally, two studies show the psychological impact on students with specific educational needs (Lavigne-Cerván et al.; Soriano-Ferrer et al.).

One especially relevant aspect is the behavioral change in technology use during the pandemic (Nieto-Escamez and Roldán-Tapia; Obrero-Gaitán et al.; Provenzi et al.; Yang et al.). Other changes in the teaching-learning process have also come about during this period (Buško and Bezinović; de la Fuente et al.; Jelińska and Paradowski; Xu et al.; Ozamiz-Etxebarria et al.).

An interesting closing paper offers the perspective of psychoeducational support that has been provided during the pandemic (Karaman et al.).

The Editors of this Research Topic wish to explicitly acknowledge the commitment of the publishers in producing this monograph free of charge. We also acknowledge the authors whose contributions make up the content.

## Author contributions

All authors listed have made a substantial, direct, and intellectual contribution to the work and approved it for publication.

## Funding

This study was supported by R&D Project PGC2018-094672-B-I00, University of Navarra, Ministry of Education and Science (Spain), and the European Social Fund (EU), R&D Project UAL18- SEJ-DO31-A-FEDER, and University of Almería (Spain) and the European Social Fund (EU) (www.inetas.net).

## Conflict of interest

The authors declare that the research was conducted in the absence of any commercial or financial relationships that could be construed as a potential conflict of interest.

## Publisher's note

All claims expressed in this article are solely those of the authors and do not necessarily represent those of their affiliated organizations, or those of the publisher, the editors and the reviewers. Any product that may be evaluated in this article, or claim that may be made by its manufacturer, is not guaranteed or endorsed by the publisher.

